# Immunohistochemical Loss of MTAP as a Diagnostic and Prognostic Surrogate of *CDKN2A/B* Homozygous Deletion: A Narrative Review

**DOI:** 10.3390/diagnostics16132069

**Published:** 2026-07-01

**Authors:** Serena Salzano, Rosario Caltabiano, Andrea Palicelli, Maurizio Zizzo, Massimiliano Fabozzi, Nektarios Koufopoulos, Ioannis Boutas, Gerardo Cazzato, Magda Zanelli, Giuseppe Broggi

**Affiliations:** 1 Department of Medical and Surgical Sciences and Advanced Technologies “G.F. Ingrassia” Anatomic Pathology, University of Catania, 95123 Catania, Italy; sere.salzano@gmail.com (S.S.); rosario.caltabiano@unict.it (R.C.); giuseppe.broggi@phd.unict.it (G.B.); 2 Pathology Unit, Azienda USL-IRCCS di Reggio Emilia, 42123 Reggio Emilia, Italy; andrea.palicelli@ausl.re.it; 3 Surgical Oncology Unit, Azienda USL-IRCCS di Reggio Emilia, 42122 Reggio Emilia, Italy; maurizio.zizzo@ausl.re.it (M.Z.); massimiliano.fabozzi@ausl.re.it (M.F.); 4 Second Department of Pathology, Medical School, National and Kapodistrian University of Athens, Attikon University Hospital, 12462 Athens, Greece; nkoufo@med.uoa.gr (N.K.); iboutas@med.uoa.gr (I.B.); 5 Department of Precision and Regenerative Medicine and Ionian Area (DiMePRe-J), Section of Molecular Pathology, University of Bari “Aldo Moro”, 70121 Bari, Italy; gerardo.cazzato@uniba.it

**Keywords:** MTAP immunohistochemistry, *CDKN2A/B* homozygous deletion, 9p21 chromosomal loss, PRMT5/MAT2A synthetic lethality, prognostic biomarker

## Abstract

Methylthioadenosine phosphorylase (MTAP) immunohistochemistry (IHC) has emerged as a valuable diagnostic, prognostic, and therapeutic biomarker in modern oncologic pathology, primarily serving as a surrogate for *CDKN2A/B* homozygous deletion due to their close genomic proximity at chromosome 9p21. This review aims to systematically evaluate the clinical utility, diagnostic accuracy, and technical limitations of MTAP IHC across a diverse spectrum of human malignancies, while contextualizing its role within current molecular testing algorithms. We first examine the established diagnostic and grading performance of MTAP loss in central nervous system neoplasms and thoracic tumors, particularly malignant pleural mesothelioma, followed by an analysis of its emerging prognostic value in gastrointestinal, cutaneous, and genitourinary malignancies. Furthermore, we discuss the therapeutic implications of MTAP deficiency, focusing on the biological consequences of methylthioadenosine accumulation and the resulting synthetic vulnerabilities in the PRMT5/MAT2A pathway. By synthesizing diagnostic precision, prognostic relevance, and translational therapeutic insights, this review provides a comprehensive framework for integrating MTAP IHC into routine surgical pathology workflows and personalized oncology.

## 1. Introduction

**Figure 1:**
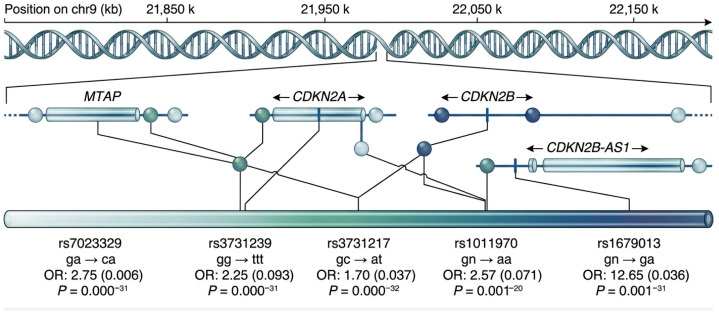
The genomic architecture of the 9p21 locus and the precise relative positions of MTAP and CDKN2A/B, a schematic representation.

Methylthioadenosine phosphorylase (MTAP) has progressively emerged as a central biomarker in modern oncologic pathology, reflecting one of the most recurrent and biologically consequential genomic events in human cancer: deletion of the chromosome 9p21 region (Figure 1). This locus harbors the tumor suppressor genes *CDKN2A* and *CDKN2B*, which encode critical regulators of cell-cycle control, and its homozygous deletion represents a defining alteration across multiple tumor types [1]. Because the MTAP gene lies immediately adjacent to *CDKN2A/B* on 9p21, these deletions frequently encompass MTAP, resulting in loss of protein expression that can be readily detected by immunohistochemistry (IHC). This genomic proximity has transformed MTAP from a metabolic enzyme of biochemical interest into a practical, morphology-preserving surrogate marker for *CDKN2A/B* homozygous deletion [2]. In recent years, the application of MTAP IHC has expanded across central nervous system, thoracic, gastrointestinal, cutaneous, genitourinary, and other malignancies, where it serves not only as a diagnostic adjunct but also as a powerful prognostic and potentially predictive biomarker [3].

In neuro-oncologic practice, assessment of 9p21 deletion status is integral to the molecular classification of diffuse gliomas, including the upgrading of IDH-mutant astrocytomas to WHO grade 4 even in the absence of necrosis or microvascular proliferation. Within this context, MTAP IHC provides a rapid and cost-effective alternative to molecular assays such as fluorescence in situ hybridization (FISH) or next-generation sequencing (NGS), particularly in resource-limited settings. Comparable diagnostic and prognostic implications have been documented in pleomorphic xanthoastrocytoma, meningioma, and pediatric high-grade gliomas. In thoracic pathology, MTAP loss has become a key ancillary marker in malignant pleural mesothelioma, assisting in the distinction between malignant and reactive mesothelial proliferations and complementing BAP1 evaluation [1]. Parallel developments in lung carcinoma have highlighted MTAP deficiency as a defining feature of aggressive molecular subsets characterized by genomic instability and therapeutic resistance. Beyond its diagnostic role, MTAP loss carries profound biological implications: the absence of MTAP activity leads to intracellular accumulation of methylthioadenosine (MTA), which interferes with arginine methyltransferase activity and generates a state of synthetic vulnerability centered on the PRMT5/MAT2A axis, thereby providing a mechanistic rationale for targeted therapeutic strategies [4].

The objective of this review is to provide a comprehensive and integrative overview of MTAP immunohistochemistry as a surrogate marker for *CDKN2A/B* homozygous deletion across multiple tumor types, critically analyzing its diagnostic accuracy, technical limitations, and prognostic significance.

While the clinical utility of MTAP immunohistochemistry extends across a broad spectrum of human neoplasms, Table 1 highlights the principal tumor types where its diagnostic accuracy, prognostic relevance, and correlation with *CDKN2A/B* deletion have been most extensively validated in the current literature.

Furthermore, Table 1 summarizes the various functions and applications of MTAP immunohistochemistry across different tumor types, as well as its prognostic, diagnostic, and therapeutic significance. Furthermore, this review aims to contextualize MTAP deficiency within its broader biological framework, examining its role in tumor progression, metabolic reprogramming, and emerging targeted therapies. By synthesizing evidence from central nervous system, thoracic, gastrointestinal, cutaneous, genitourinary, and other malignancies, we seek to delineate the current clinical applications of MTAP IHC, identify areas of controversy or variability, and highlight future directions for translational research and precision oncology.

To ensure a rigorous and transparent approach to this comprehensive narrative review, a structured literature search strategy was implemented across major electronic databases, including PubMed/MEDLINE and Scopus, from database inception through January 2026. The search methodology utilized combinations of relevant Medical Subject Headings (MeSH) terms and high-specificity keywords, including ‘methylthioadenosine phosphorylase’, ‘MTAP’, ‘immunohistochemistry’, ‘CDKN2A/B’, and ‘9p21 deletion’, combined via Boolean operators with organ-specific descriptors such as ‘glioma’, ‘mesothelioma’, ‘melanoma’, ‘pancreatic adenocarcinoma’, ‘gastrointestinal stromal tumor’, and ‘urothelial carcinoma’. Inclusion criteria targeted peer-reviewed, English-language original research articles, prospective and retrospective clinical trials, and multi-center cohort validations focused on the diagnostic accuracy, prognostic correlation, or predictive utility of MTAP immunohistochemistry in human oncology. Document screening, full-text evaluation, and data synthesis were independently performed and cross-verified by multiple co-authors to minimize individual selection bias and ensure a comprehensive, multidisciplinary integration of the current clinical evidence.

## 2. Central Nervous System Tumors

In neuro-oncological practice, the detection of *CDKN2A/B* homozygous deletion is primarily utilized for the refined classification of diffuse gliomas, being considered as molecular criterion for upgrading an *IDH*-mutant astrocytoma to WHO grade 4, even in the absence of conventional histological features like necrosis or microvascular proliferation [5]. Furthermore, in pleomorphic xanthoastrocytomas (PXAs) and certain pediatric high-grade gliomas, the identification of CDKN2A/B homozygous deletion provides essential prognostic information, often correlating with increased tumor aggressiveness and a shorter time to recurrence. The immunohistochemical loss of MTAP, as surrogate of *CDKN2A/B* homozygous deletion, has been extensively investigated across different tumor types, including astrocytoma, oligodendroglioma, PXA and meningiomas [6,41].

In this regard, our research group recently investigated whether immunohistochemistry for MTAP and p16 could serve as reliable, cost-effective surrogate markers for detecting *CDKN2A/B* homozygous deletion in a range of central nervous system tumors [6]. In a cohort of 227 patients with different tumor types, including *IDH*-wildtype glioblastomas, meningiomas, *IDH*-mutant astrocytomas, *IDH*-mutant and 1p/19q codeleted oligodendrogliomas and pleomorphic xanthoastrocytomas, loss of MTAP expression and negative p16 staining were strongly associated with *CDKN2A/B* deletion [6]. When MTAP and p16 immunohistochemistry results were combined, they demonstrated high sensitivity, specificity and predictive values for identifying cases with the deletion; furthermore, patients whose tumors showed MTAP loss, p16 negativity and *CDKN2A/B* deletion had significantly worse disease-free and overall survival [6]. The authors concluded that MTAP immunohistochemistry, particularly when interpreted alongside p16 results, is a feasible and practical surrogate for *CDKN2A/B* homozygous deletion in central nervous system tumors, and that these markers also provide meaningful prognostic information [6]. Other authors evaluated whether loss of MTAP and p16 proteins detected by immunohistochemistry could reliably indicate homozygous deletion of the *CDKN2A/B* gene locus in diffuse gliomas [4]. Across 100 glioma cases, almost all tumors that showed absence of MTAP and p16 expression corresponded to homozygous *CDKN2A/B* deletion when assessed by copy number variation plots, and the few discordant cases were confirmed as deleted by FISH analysis [4]. Furthermore, the presence of MTAP deficiency correlated with significantly shorter survival in several glioma subgroups, including IDH-mutant astrocytomas and oligodendrogliomas as well as *IDH*-wild-type gliomas, emphasizing that MTAP immunostaining is a useful, robust, low-cost adjunct for glioma diagnostics that correlates well with *CDKN2A/B* deletion and provides meaningful prognostic information, whereas p16 results should be interpreted cautiously [4]. Studies have shown that in *IDH*-mutant gliomas, absence of MTAP expression strongly correlates with *CDKN2A/B* deletion, with high sensitivity and specificity, making MTAP immunostaining a reliable, cost effective surrogate when molecular testing is unavailable or limited [7,8,9]. Clinically, tumors with MTAP loss tend to exhibit more aggressive behavior and shorter survival, highlighting its potential role not only in diagnostic and grading decisions but also in risk stratification [7,8,9]. While MTAP immunohistochemistry is robust, some intratumoral heterogeneity can occur, so interpretation alongside p16 and molecular data is often recommended for maximal accuracy [6]. MTAP loss has also emerged as an important molecular marker in meningiomas, particularly in higher grade and more aggressive tumors, serving as a surrogate for molecular testing such as FISH or copy number analysis also in this tumor type [10,11,12]. Studies have shown that MTAP loss is more frequent in WHO grade 2 and 3 meningiomas than in benign (grade 1) tumors, and correlates with more aggressive behavior [13,14]. Importantly, MTAP immunostaining can be integrated into routine histopathological evaluation to help stratify patients for closer follow-up or adjuvant therapy [10,11,12].

Regarding PXA, Vizcaino et al. PXA cases with available chromosomal microarray data to compare the presence or absence of p16 and MTAP protein expression against actual gene deletion status [15]. While p16 immunohistochemistry showed high sensitivity (~94.6%) for detecting *CDKN2A* homozygous deletion, MTAP loss alone had lower sensitivity (~73%). However, combining p16 and MTAP immunostains detected all cases with *CDKN2A* deletion, including those where p16 staining failed, the authors concluded that although MTAP IHC by itself is less sensitive, when used with p16 staining it enhances the immunohistochemical detection of *CDKN2A* homozygous deletion in PXA, offering a practical approach to surrogate molecular assessment [15].

## 3. Thoracic Tumors

MTAP deficiency is increasingly recognized as a pivotal molecular biomarker in thoracic pathology, particularly within the landscape of large cell neuroendocrine carcinoma (LCNEC) and highly aggressive subsets of non-small cell lung cancer (NSCLC) [21]. This metabolic deficiency often identifies a distinct molecular phenotype characterized by heightened genomic instability and a frequent lack of responsiveness to standard cytotoxic chemotherapy, necessitating a more nuanced diagnostic approach.

In clinical practice, the IHC evaluation of MTAP expression serves as a robust tool for the molecular stratification of these tumors, aiding in the complex differentiation between primary pulmonary neuroendocrine carcinomas and various metastatic lesions while simultaneously identifying patients who may benefit from clinical trials involving targeted metabolic inhibitors. The genomic loss of MTAP is frequently accompanied by a hyper-dependence on Protein Arginine Methyltransferase 5 (PRMT5), an enzyme that has been deeply implicated in lung cancer progression through the methylation of miR-99 family histones and the subsequent activation of oncogenic Erk1/2 and Akt signaling pathways [22].

Furthermore, PRMT5 functions as a critical inhibitor of tumor suppressor genes, specifically inducing the methylation of p53 to disrupt its pro-apoptotic capacity and promoting cyclin-dependent neoplastic growth. While historical attempts to exploit this metabolic vulnerability with agents like L-alanosine in the 1980s failed to produce clinically significant outcomes in NSCLC or mesothelioma, modern pharmacological research has demonstrated that selective PRMT5 inhibitors, such as AMI-1 and GSK591, can effectively promote apoptosis and enhance chemosensitivity [23]. Notably, the inhibition of PRMT5 has been shown to cause pronounced cell cycle arrest in lung adenocarcinoma lines, a therapeutic effect that appears significantly amplified when combined with platinum-based agents such as cisplatin [23]. Consequently, the loss of MTAP not only sensitizes malignant cells to these targeted inhibitors but also suggests a need for tailored immunotherapy and combination strategies to improve the clinical paradigm for patients harboring these specific molecular alterations [24].

Chapel et al. examined the relationship between MTAP protein expression and copy number changes in the MTAP and *CDKN2A* genes in malignant pleural mesothelioma [16]. Because *MTAP* and *CDKN2A* are located adjacent to each other on chromosome 9p21, deletions in this region—particularly of CDKN2A—are common in mesothelioma and are diagnostically significant. The authors analyzed tumor samples to compare MTAP protein expression, assessed by immunohistochemistry, with underlying gene copy number status [16]. They found a strong correlation between loss of MTAP protein expression and deletion of both *MTAP* and *CDKN2A* genes. In most cases, tumors with *CDKN2A* deletions also showed absence of MTAP protein. The findings suggested that immunohistochemical detection of MTAP loss could serve as a reliable surrogate marker for *CDKN2A* deletion, providing a practical and accessible diagnostic tool in settings where molecular testing methods such as fluorescence in situ hybridization are not readily available [16]. Lynggård et al. evaluated whether loss of BAP1 and MTAP expression, detected by IHC, can reliably distinguish malignant mesothelioma from reactive mesothelial proliferations using cytologic specimens from serous effusions (such as pleural fluid), comparing these results to biopsy tissue [17]. Using samples from 162 patients with mesothelioma (71 cytologic, 91 histologic) and 20 benign controls, the authors found that loss of BAP1 and/or MTAP expression showed excellent specificity (100%) and high sensitivity (~79–80%) for identifying mesothelioma in both cytology and biopsy specimens, with no significant difference between effusion cytology and tissue sections [17]. There was complete concordance in marker expression between paired cytology and histology from the same patients. The combined loss of either marker performed well across mesothelioma subtypes (epithelioid, biphasic, sarcomatoid). The findings suggest that assessing BAP1 and MTAP by IHC in effusion cytology can effectively differentiate malignant from benign mesothelial proliferations, offering a less invasive diagnostic adjunct comparable to biopsy results, though normal expression of both markers does not rule out mesothelioma due to the lower sensitivity [17]. Another study evaluated the usefulness and limitations of using MTAP IHC as a diagnostic aid in difficult cases of desmoplastic mesothelioma and sarcomatoid pleural mesothelioma with desmoplastic features [18]. These subtypes of malignant mesothelioma can be particularly challenging to diagnose because their spindle-cell and fibrous/desmoplastic morphology can resemble benign fibrous tissues or reactive conditions. While genomic-based ancillary assays, including IHC for MTAP and IHC for other markers such as BAP1, are commonly employed to distinguish mesothelioma from reactive mesothelial proliferations, the authors found that MTAP IHC alone has limitations in these specific mesothelioma subtypes [18]. In desmoplastic and sarcomatoid cases, loss of MTAP expression was not consistently observed, reducing its diagnostic sensitivity in this setting despite its utility in other mesothelioma forms [18]. The study emphasized that while MTAP IHC could be a helpful tool in the diagnostic panel for mesothelioma overall, its performance was less reliable for desmoplastic and sarcomatoid variants, and should be interpreted cautiously and in conjunction with other markers and clinical/pathologic context [18]. Febres-Aldana et al. evaluated how well different laboratory methods detected the loss of MTAP [19]. The researchers compared two monoclonal antibodies (EPR6893 and 1813) used in MTAP IHC and measured how their staining results aligned with 9p21 copy number status determined by targeted NGS and, in discrepant cases, *CDKN2A* FISH [19]. They found that antibody 1813 produced stronger and more specific staining with fewer equivocal results than EPR6893. When IHC using antibody 1813 was compared with combined molecular assessments (NGS and FISH), it showed high sensitivity, specificity, and overall accuracy for detecting homozygous 9p21 deletions, indicating very good agreement between MTAP IHC and genomic status [19]. The authors concluded that MTAP IHC, especially with antibody 1813, could reliably indicate underlying 9p21 deletion and was complementary to molecular assays, particularly useful in samples with low tumor purity or in resource-limited settings [19].

Conversely, Quaranta et al. speculated whether immunohistochemical loss of MTAP protein can reliably stand in for detection of *CDKN2A* gene homozygous deletion in peritoneal mesothelioma [20]; they analyzed 39 formalin-fixed paraffin-embedded peritoneal mesothelioma samples by FISH to assess *CDKN2A* status and by IHC to evaluate MTAP expression using two positivity thresholds (≥1% and ≥30% of cells) [20]. They also included 14 benign peritoneal lesions as controls, all of which retained MTAP expression and showed no *CDKN2A* deletion; among the tumour cases, *CDKN2A* homozygous deletion was detected in the majority, but loss of MTAP expression was observed in only a subset [20]. Statistical analysis revealed very low and non-significant agreement between MTAP IHC results and *CDKN2A* deletion status, and receiver operating characteristic analysis showed that MTAP IHC had poor ability to discriminate between tumours with and without *CDKN2A* deletion [20]. There was a statistically significant discordance between the two methods. Accordingly, the authors conclude that, unlike in pleural mesothelioma, MTAP immunohistochemistry did not reliably reflect *CDKN2A* homozygous deletion in peritoneal mesothelioma and therefore cannot be used as a substitute for direct molecular testing in this tumour type, suggesting biological or pathogenetic distinctions between peritoneal and pleural mesotheliomas [20].

## 4. Gastrointestinal Tumors

In the context of gastrointestinal oncology, MTAP IHC has emerged as a multifaceted tool, functioning simultaneously as a diagnostic adjunct and a potential predictive biomarker for emerging metabolic therapies. In pancreatic ductal adenocarcinoma (PDAC), the loss of MTAP expression is a frequent molecular event, typically representing a late-stage genetic alteration that correlates with heightened chemoresistance and overall poor clinical outcomes. The application of IHC is particularly advantageous in the setting of PDAC due to the tumor’s hallmark histomorphology, characterized by low neoplastic cellularity embedded within a prominent, dense desmoplastic stroma [25]. While traditional molecular assays, such as FISH or NGS, often struggle to provide definitive results in samples with a high ratio of non-neoplastic to malignant cells, IHC facilitates the direct visualization of protein loss specifically within the epithelial compartment. By maintaining morphological integrity, IHC bypasses the limitations of “bulk” genetic testing, which frequently requires high tumor purity to avoid false-negative results for homozygous deletions [26].

Regarding the therapeutic landscape, current evidence suggests that approximately one-third of pancreatic adenocarcinomas harbor MTAP deficiency. This loss creates a metabolic “Achilles’ heel” by forcing the cells to rely exclusively on the de novo purine biosynthetic pathway for survival. Consequently, the identification of MTAP-deficient cohorts through highly sensitive and specific IHC assays enables the precise selection of patients who may benefit from targeted inhibitors of this pathway, such as MAT2A or PRMT5 inhibitors, which exploit the principle of synthetic lethality [27].

In gastrointestinal stromal tumors (GISTs), loss of MTAP expression has been identified in a distinct subset of biologically aggressive cases and is widely regarded as a surrogate marker of p16/*CDKN2A* pathway inactivation, carrying independent negative prognostic significance [28]. Clinically, recognition of MTAP-deficient GISTs is highly relevant for risk stratification, as these tumors are typically characterized by increased mitotic activity and a markedly higher likelihood of local recurrence and distant metastasis. Homozygous deletion of *MTAP* has demonstrated strong concordance with loss of protein immunoexpression, supporting this genomic alteration as the principal mechanism underlying MTAP deficiency in GISTs; however, alternative genetic events contributing to MTAP downregulation, including point mutations or small deletions involving more than five exons and/or the MTAP promoter region, cannot be entirely excluded. The study by Huang et al. further demonstrated that homozygous *MTAP* deletion correlates with larger tumor size, higher mitotic count, increased Ki-67 proliferation index, and higher NIH risk category, and independently predicts significantly worse disease-free survival, conferring more than a twofold increased risk of recurrence [29]. Notably, *MTAP* promoter hypermethylation, although observed in a small subset of cases, was detected exclusively in non–high-risk tumors, suggesting that this epigenetic alteration may occur at earlier stages of tumorigenesis, similar to what has been described in precancerous cirrhotic liver tissue [30]. Moreover, GISTs harboring non-homozygous *MTAP* deletions exhibited an indolent clinical course comparable to that of MTAP-expressing tumors, in contrast to the aggressive behavior observed in cases with homozygous deletion, implying that the timing and mechanism of MTAP inactivation during GIST evolution may influence its prognostic impact. Overall, homozygous *MTAP* deletion represents the predominant mechanism driving reduced MTAP protein expression and is not only associated with adverse clinico-pathologic parameters but also exerts an independent prognostic effect, underscoring its pivotal role in GIST progression.

Furthermore, the dual immunohistochemical evaluation of p16 and MTAP has been validated as a robust surrogate marker for *p16INK4A/CDKN2A* homozygous deletions across various gastrointestinal malignancies. This combined approach offers a practical, cost-effective, and rapid alternative to complex genetic sequencing in routine clinical practice. By providing spatial context to molecular loss, this IHC-based strategy ensures that even in biopsies with scant tumor tissue, the clinician can accurately determine the molecular profile necessary to guide both prognostic assessment and the selection of personalized, metabolism-targeted treatment regimens [31].

## 5. Skin Tumors

In cutaneous malignancies, particularly within the evolving landscape of melanoma diagnostics, the loss of MTAP expression has been identified as a definitive hallmark of clonal evolution, specifically marking the critical transition from in situ lesions to invasive and metastatic disease states. The deletion of the 9p21 locus represents a pivotal genomic event in melanocytic progression, where the immunohistochemical loss of MTAP correlates significantly with aggressive clinical parameters, including increased Breslow thickness and elevated mitotic indices, thereby providing essential prognostic stratification. This utility extends into the diagnostic evaluation of spitzoid neoplasms, where MTAP loss serves as a vital tool in identifying tumors with latent malignant potential that might otherwise present as morphologically ambiguous or borderline lesions [32].

While complete loss of p16 expression is frequently observed in the majority of melanomas harboring even a single-copy deletion of *CDKN2A*, contemporary literature suggests that MTAP expression is typically maintained or exhibits a characteristic mosaic pattern in the context of hemizygous *CDKN2A* loss, a distinction that underscores the value of MTAP as a highly specific surrogate marker for the homozygous deletion of the *CDKN2A* locus [33,34].

Furthermore, the absence of MTAP protein expression is strongly associated with the homozygous deletion of the MTAP locus itself, a molecular vulnerability that creates a unique metabolic context characterized by the accumulation of MTA. This metabolic shift induces a state of collateral lethality by conferring a cellular dependency on the PRMT5 pathway, which has catalyzed the development of novel therapeutic strategies targeting the PRMT5/MAT2A axis in MTAP-deficient solid tumors. Beyond these applications, MTAP status is emerging as a promising predictive biomarker for sensitivity to interferon therapy, potentially expanding its clinical relevance from a purely diagnostic tool to a predictive one, provided that further validation is achieved in broader prospective cohorts [4].

From a technical standpoint, MTAP is increasingly favored due to its exceptional specificity and binary staining profile; unlike p16, which often yields complex or equivocal patchy patterns, MTAP loss typically manifests as a definitive absence of cytoplasmic staining in neoplastic cells while maintaining robust expression in internal non-neoplastic controls, such as stromal fibroblasts and inflammatory infiltrates. Ultimately, the integrated assessment of the p16/MTAP axis, when correlated with molecular findings, offers a sophisticated framework for navigating the diagnostic, prognostic, and translational complexities of the melanocytic genomic landscape, though current therapeutic implications should be interpreted as emerging opportunities rather than established routine selection criteria for borderline cases [35].

## 6. Other Tumors

In the realm of multi-organ diagnostic pathology, the utility of MTAP IHC has expanded significantly, serving as a critical indicator of genomic instability and a surrogate for complex molecular alterations. Within the gastrointestinal and pancreatobiliary tracts, MTAP deficiency is a recurrent finding in intrahepatic cholangiocarcinomas, PDAC, and esophageal squamous cell carcinomas. In these malignancies, the loss of MTAP expression is not merely a molecular bystander but typically signals an advanced pathological stage and an unfavorable prognosis, often correlating with aggressive invasive patterns and resistance to conventional therapeutic regimens [42].

In the genitourinary system, MTAP loss is increasingly recognized as a hallmark of aggressive renal cell carcinoma (RCC) variants and muscle-invasive urothelial carcinoma (UC).

Decreased expression of MTAP has been observed in RCC tissues, where it significantly correlates with higher histological grades and shortened overall survival. Functional genetic studies indicate that MTAP expression exerts a suppressive effect on the epithelial–mesenchymal transition (EMT), as well as on the invasive and migratory capabilities of RCC cells [36]. Notably, MTAP knockout experiments reveal a distinctive biochemical shift characterized by a reduction in global protein methylation levels with a concomitant increase in tyrosine phosphorylation, suggesting a complex interplay between metabolic deficiency and oncogenic signaling pathways [37].

The pathogenesis of UC is frequently characterized by the HD of the chromosomal region 9p21.3, a critical genetic event that results in the contiguous loss of several key tumor suppressor genes, most notably *CDKN2A* and *MTAP*. Since this protein deficiency is predominantly homogeneous in advanced urothelial carcinoma, IHC assessment of MTAP has emerged as a highly validated and accessible surrogate marker, offering near-perfect sensitivity and specificity for the detection of 9p21.3 HD [38]. This diagnostic reliability is particularly significant as it identifies a substantial subset of patients with invasive bladder tumors who may derive profound clinical benefit from emerging therapeutic strategies specifically designed to exploit MTAP-deficient metabolic vulnerabilities [39].

Beyond its role as a prognostic proxy, MTAP IHC functions as a vital diagnostic tool for the differential diagnosis of ambiguous bladder specimens, effectively distinguishing malignant urothelial neoplasia from its benign mimic, reactive urothelium. Furthermore, the application of IHC provides a unique spatial perspective, allowing researchers and clinicians to investigate clinicopathological associations and the intricate inter-tumoral and intra-tumoral heterogeneity of 9p21.3 HD across the entire clinical spectrum of urothelial manifestations, from localized, non-invasive disease to advanced metastatic progression [38].

Furthermore, research by Nilforoushan et al. has demonstrated a significant correlation between the loss of MTAP expression and adverse clinical behavior in ovarian borderline serous tumors, suggesting that this metabolic deficiency may serve as a critical indicator of increased malignancy. These findings underscore the necessity for expanded longitudinal studies across larger cohorts to formally validate the utility of immunohistochemical MTAP staining as a reliable prognostic biomarker in these specific neoplasms. Integrating this evidence with the established role of MTAP in thoracic pathology reinforces the broader clinical significance of this marker in identifying high-risk phenotypes and guiding more aggressive management strategies across diverse oncological contexts [40].

Finally, MTAP IHC has established itself as a cornerstone in thoracic pathology, specifically for the diagnosis of malignant mesothelioma. One of the most challenging distinctions in surgical pathology is the separation of malignant mesothelial proliferations from reactive mesothelial hyperplasia. Since MTAP (and *CDKN2A*) loss is highly specific for malignancy in this context, the preservation of its expression in reactive cells provides an objective, reliable parameter to confirm a diagnosis of mesothelioma. By integrating MTAP IHC into the routine diagnostic algorithm, pathologists can enhance diagnostic specificity, facilitating earlier clinical intervention and more accurate risk stratification for these highly aggressive tumors.

## 7. Discussion

The broad applicability of MTAP immunohistochemistry across organ systems reflects the convergence of practical diagnostic utility and mechanistic oncologic relevance. In central nervous system tumors, multiple studies have demonstrated a strong concordance between MTAP protein loss and *CDKN2A*/B homozygous deletion, particularly in IDH-mutant astrocytomas and oligodendrogliomas. The integration of MTAP and p16 IHC enhances sensitivity and specificity for detecting 9p21 deletion, with combined assessment achieving high predictive value in several cohorts. Importantly, MTAP loss correlates with significantly shorter disease-free and overall survival, underscoring its independent prognostic impact. Nevertheless, occasional intratumoral heterogeneity and rare discordant cases highlight the necessity of interpreting IHC findings within a comprehensive molecular and histopathological framework [6].

In thoracic malignancies, MTAP IHC demonstrates both diagnostic precision and biological depth. In pleural mesothelioma, loss of MTAP expression shows strong concordance with *CDKN2A* deletion and provides excellent specificity when distinguishing malignant mesothelioma from reactive mesothelial hyperplasia, even in cytologic specimens derived from serous effusions. When combined with BAP1 immunostaining, the specificity for malignancy approaches 100%, although sensitivity remains imperfect, and preserved expression does not exclude disease. Importantly, the diagnostic performance of MTAP IHC is more limited in desmoplastic and sarcomatoid variants and appears unreliable in peritoneal mesothelioma, suggesting site-specific or subtype-specific pathogenetic differences. In non–small cell lung carcinoma and large cell neuroendocrine carcinoma, MTAP deficiency delineates molecularly aggressive subgroups with heightened genomic instability and reduced responsiveness to conventional chemotherapy, while simultaneously identifying tumors potentially susceptible to PRMT5-targeted therapies [35].

In gastrointestinal oncology, the advantages of MTAP IHC are particularly evident in pancreatic ductal adenocarcinoma, where dense desmoplastic stroma often compromises the analytical sensitivity of bulk genomic assays. Immunohistochemistry preserves tissue architecture and enables selective evaluation of tumor cells within a heterogeneous microenvironment, mitigating the risk of false-negative results in low-purity specimens. Approximately one-third of pancreatic adenocarcinomas exhibit MTAP deficiency, a late-stage event associated with adverse prognosis and chemoresistance. Similar prognostic associations have been documented in gastrointestinal stromal tumors, in which homozygous MTAP deletion correlates with increased tumor size, higher mitotic activity, elevated proliferation indices, and significantly reduced disease-free survival [42].

In cutaneous melanoma, MTAP loss marks a critical step in clonal progression and correlates with aggressive features such as increased Breslow thickness and mitotic rate. Unlike p16, which may demonstrate patchy or equivocal staining and may be lost in hemizygous deletions, MTAP typically exhibits a binary staining pattern with strong internal controls, enhancing interpretive reliability and specificity for homozygous *CDKN2A* deletion. In genitourinary tumors, particularly muscle-invasive urothelial carcinoma, MTAP loss is highly concordant with 9p21.3 homozygous deletion and is remarkably homogeneous in advanced disease, making it a robust surrogate for molecular testing and a promising stratification marker for emerging metabolism-targeted therapies [33,34].

Across tumor types, technical considerations remain critical. Antibody selection, staining protocols, positivity thresholds, and the presence of internal non-neoplastic controls significantly influence interpretive accuracy. Studies comparing monoclonal antibodies have demonstrated variability in sensitivity and specificity, emphasizing the need for standardized validation. Furthermore, while MTAP IHC provides high concordance with genomic assays in many contexts, it should not universally replace molecular testing, particularly in tumor subtypes where discordance has been documented.

## 8. Knowledge Gaps and Future Perspectives

Despite the expanding clinical implementation of MTAP immunohistochemistry, several critical knowledge gaps persist that warrant dedicated future investigation. First, while the concordance between MTAP protein loss and homozygous CDKN2A/B deletion is well-characterized in major tumor types like glioblastomas and pleural mesotheliomas, there is a distinct lack of large-scale, multi-center prospective studies validating its predictive accuracy in rarer or histologically challenging variants, such as peritoneal, desmoplastic, and sarcomatoid neoplasms. Furthermore, the precise epigenetic and transcriptional mechanisms driving non-deletion-mediated MTAP downregulation, including promoter hypermethylation or microRNA regulation, remain poorly understood and require comprehensive multi-omic profiling to prevent diagnostic misclassification. Another major limitation in the current literature is the absence of standardized, universally accepted scoring systems and diagnostic cut-offs for MTAP immunoreactivity, as different studies employ highly variable positivity thresholds ranging from a strict binary loss to specific semi-quantitative percentages. Future research should focus on cross-institutional harmonization trials to establish robust guidelines for digital image analysis and automated scoring, minimizing inter-pathologist variability. Clinically, the most pressing objective for prospective trials is to determine whether MTAP deficiency, as verified by immunohistochemistry, can reliably serve as a predictive biomarker for patient stratification in emerging clinical trials evaluating MAT2A and PRMT5 inhibitors. Longitudinal studies investigating the dynamic clonal evolution of MTAP loss from primary tumors to metastatic sites are crucial to uncovering mechanisms of metabolic adaptation and therapeutic resistance, ultimately bridging the gap between routine morphological evaluation and precision metabolic oncology.

## 9. Limitations

Although MTAP immunohistochemistry represents a robust and readily accessible diagnostic surrogate, several technical and biological limitations necessitate a cautious interpretation of its results in clinical practice. A primary diagnostic challenge stems from the occurrence of subtype-specific discordances, where MTAP protein expression does not perfectly align with the underlying *CDKN2A/B* genomic status. For instance, while the correlation remains exceptionally high in central nervous system tumors and malignant pleural mesotheliomas, its reliability drops significantly in specific histological variants, such as desmoplastic, sarcomatoid, and peritoneal mesotheliomas, where structural genomic heterogeneity or distinct pathogenetic mechanisms can lead to false-negative or false-positive interpretations [18].

Furthermore, technical variables inherent to immunohistochemical assays introduce potential confounding factors, including variations in antibody clones, tissue fixation protocols, and the subjective nature of semi-quantitative scoring systems among different pathologists. The presence of non-neoplastic stromal cells, infiltrating lymphocytes, or reactive mesothelial elements within low-tumor-purity specimens can also mask the homozygous deletion by providing a false-positive internal positive control, thereby requiring a high degree of morphological expertise to isolate tumor-specific signals. Lastly, because MTAP loss can occasionally occur independently of *CDKN2A/B* deletions through epigenetic silencing, promoter hypermethylation, or distinct focal mutational events, relying solely on protein expression may overlook complex genomic landscapes. These limitations underscore the fact that while MTAP IHC is an invaluable screening tool, it cannot entirely replace complementary molecular testing methods, such as FISH or NGS, which remain essential in diagnostically ambiguous or histologically challenging clinical scenarios.

## 10. Conclusions

In conclusion, MTAP immunohistochemistry represents a major advancement in translational oncology, effectively bridging the gap between sophisticated molecular genetics and routine, morphology-driven surgical pathology. By serving as a highly reliable, cost-effective, and rapid surrogate marker for *CDKN2A/B* homozygous deletion, MTAP evaluation provides clinicians with vital diagnostic and prognostic clarity across a rapidly expanding spectrum of human malignancies. Rather than replacing molecular testing, this immunohistochemical assay acts as an invaluable screening tool that optimizes diagnostic workflows, particularly in low-resource settings or when dealing with low-tumor-purity specimens that challenge traditional genomic assays. Looking ahead, the clinical relevance of MTAP is poised to undergo a profound shift from a purely diagnostic and prognostic proxy to a predictive biomarker for personalized medicine. As novel targeted therapies designed to exploit the synthetic lethality of the PRMT5/MAT2A axis continue to mature in clinical trials, the accurate immunohistochemical assessment of MTAP status will become essential for patient stratification. To fully realize this potential, future efforts must focus on cross-institutional standardization of scoring criteria and the integration of digital pathology to minimize inter-observer variability. Ultimately, the routine implementation of MTAP immunohistochemistry underscores the power of metabolic biomarkers in precision oncology, offering a multi-dimensional framework that enhances diagnostic precision while opening new therapeutic avenues for patients harboring aggressive, deletion-driven tumors.

## Figures and Tables

**Table 1:** Comprehensive reference framework for MTAP immunohistochemistry in diagnostic pathology.

Tumor Type	Clinical Application	Molecular Significance	Strength of Evidence & Concordance	Key Diagnostic Limitations	Recommended Confirmatory Assays	References
**Diffuse Gliomas (IDH-mutant & wildtype)**	Molecular grading and classification; upgrading to WHO grade 4.	Surrogate for contiguous homozygous *CDKN2A/B* deletion.	High; strong concordance (>90%) verified by CNV plots and FISH.	Intratumoral heterogeneity may cause focal staining variations.	FISH or NGS in case of equivocal/patchy staining.	[4,5,6,7,8,9]
**Meningioma**	Identification of aggressive high-grade phenotypes (WHO grade 2/3).	Proxy for genomic structural changes and *CDKN2A/B* loss.	Moderate to High; correlates with recurrence and grading.	Stromal and inflammatory background cells can mask real loss.	Copy number analysis or FISH for targeted risk stratification.	[10,11,12,13,14]
**Pleomorphic Xanthoastrocytoma**	Diagnostic confirmation and risk stratification.	Surrogate for homozygous 9p21.3 deletions.	Moderate; lower sensitivity (~73%) when used as an isolated marker.	Lower single-marker sensitivity requires combined panels.	Combined MTAP and p16 IHC panel; microarray validation.	[15]
**Pleural Mesothelioma**	Differentiation between malignant and reactive mesothelial hyperplasia.	Highly specific indicator of somatic malignancy.	High; absolute specificity (100%) and high concordance with FISH.	Reduced sensitivity in desmoplastic and sarcomatoid variants.	Combined MTAP and BAP1 IHC; FISH in challenging cases.	[16,17,18,19]
**Peritoneal Mesothelioma**	Potential diagnostic adjunct.	Surrogate for structural 9p21 alterations.	Low; statistically non-significant correlation with *CDKN2A* status.	Extremely high rates of biological and technical discordance.	Direct molecular testing (FISH) is mandatory; IHC not recommended.	[20]
**Lung Carcinoma**	Molecular stratification and therapeutic target selection.	Marker for localized genomic instability and PRMT5 dependence.	Moderate to High; defines distinct aggressive molecular subsets.	High background signaling in inflammatory tumor microenvironment.	NGS panel or FISH to confirm homozygous deletion status.	[21,22,23,24]
**Pancreatic Ductal Adenocarcinoma**	Diagnostic adjunct and screening for targeted metabolic trials.	Indicator of late-stage genetic progression and purine path dependence.	High; observed loss in approximately one-third of clinical cases.	Intense desmoplastic stroma requires strict morphologic screening.	Digital image analysis or microdissection-assisted NGS.	[25,26,27]
**Gastrointestinal Stromal Tumors**	Risk stratification and prediction of local/distant recurrence.	Surrogate for structural p16/*CDKN2A* pathway inactivation.	High; homozygous loss correlates with aggressive clinical parameters.	Epigenetic hypermethylation or non-homozygous deletions limit binary scoring.	Ki-67 proliferation index correlation; FISH analysis.	[28,29,30,31]
**Cutaneous Melanoma & Spitzoid Lesions**	Assessment of clonal progression and ambiguous borderline lesions.	Marker of transition from in situ to invasive metastatic disease.	High; distinct binary profile compared to patchy p16 staining.	Hemizygous deletions can result in retained mosaic patterns.	Integrated p16/MTAP dual-IHC axis evaluation; NGS sequencing.	[4,32,33,34,35]
**Renal Cell & Urothelial Carcinoma**	Differential diagnosis and identification of advanced muscle-invasive disease.	Proxy for contiguous 9p21.3 homozygous deletion.	High; near-perfect sensitivity for homogeneous advanced stages.	Intra-tumoral structural heterogeneity across clinical spectrum.	Confirmatory genomic sequencing or FISH for targeted trial selection.	[36,37,38,39]
**Ovarian Borderline Serous Tumors**	Prognostic stratification of high-risk malignant phenotypes.	Indicator of adverse clinical behavior and metabolic shift.	Moderate; requires further validation in larger longitudinal cohorts.	Limited dataset availability in current multi-center trials.	Close clinical follow-up; longitudinal profiling.	[40]

## Data Availability

No new data were created or analyzed in this study. Data sharing is not applicable to this article.

## Abbreviations

The following abbreviations are used in this manuscript:

MTAP	Methylthioadenosine phosphorylase
CDKN2A	Cyclin-dependent kinase inhibitor 2A
CDKN2B	Cyclin-dependent kinase inhibitor 2B
IHC	Immunohistochemistry
CNS	Central nervous system
IDH	Isocitrate dehydrogenase
WHO	World Health Organization
FISH	Fluorescence in situ hybridization
NGS	Next-generation sequencing
PXA	Pleomorphic xanthoastrocytoma
LCNEC	Large cell neuroendocrine carcinoma
NSCLC	Non-small cell lung cancer
PRMT5	Protein arginine methyltransferase 5
MTA	Methylthioadenosine
MAT2A	Methionine adenosyltransferase 2A
Erk1/2	Extracellular signal-regulated kinases 1 and 2
Akt	Protein kinase B
PDAC	Pancreatic ductal adenocarcinoma
GIST	Gastrointestinal stromal tumor
Ki-67	Kiel-67 proliferation index
NIH	National Institutes of Health
RCC	Renal cell carcinoma
EMT	Epithelial–mesenchymal transition
UC	Urothelial carcinoma
HD	Homozygous deletion
